# Non-Conscious Processing of Motion Coherence Can Boost Conscious Access

**DOI:** 10.1371/journal.pone.0060787

**Published:** 2013-04-09

**Authors:** Lisandro Kaunitz, Alessio Fracasso, Angelika Lingnau, David Melcher

**Affiliations:** 1 Center for Mind/Brain Sciences (CIMeC), University of Trento, Trento, Italy; 2 Center for System Neurosciences, University of Leicester, Leicester, United Kingdom; 3 Helmholtz Institute, Department of Experimental Psychology, Utrecht University, Utrecht, The Netherlands; 4 Department of Cognitive Sciences, University of Trento, Trento, Italy; University of Rome, Italy

## Abstract

Research on the scope and limits of non-conscious vision can advance our understanding of the functional and neural underpinnings of visual awareness. Here we investigated whether distributed local features can be bound, outside of awareness, into coherent patterns. We used continuous flash suppression (CFS) to create interocular suppression, and thus lack of awareness, for a moving dot stimulus that varied in terms of coherence with an overall pattern (radial flow). Our results demonstrate that for radial motion, coherence favors the detection of patterns of moving dots even under interocular suppression. Coherence caused dots to break through the masks more often: this indicates that the visual system was able to integrate low-level motion signals into a coherent pattern outside of visual awareness. In contrast, in an experiment using meaningful or scrambled biological motion we did not observe any increase in the sensitivity of detection for meaningful patterns. Overall, our results are in agreement with previous studies on face processing and with the hypothesis that certain features are spatiotemporally bound into coherent patterns even outside of attention or awareness.

## Introduction

One of the most striking aspects of visual perception is visual awareness, the subjective experience that our brain creates from the information impinging on our retinas. Defined as “the minimal set of neuronal events and mechanisms jointly sufficient for a specific conscious percept” [1], characterizing the neural correlates of awareness remains one of the greatest puzzles of visual neuroscience. Currently, an open question is to what extent the visual system can process and interpret information of non-conscious stimuli. Where and how are non-conscious stimuli processed in the brain? Studies employing stimuli which are suppressed from awareness, such as during interocular rivalry [2], provide one way to experimentally demonstrate non-conscious processing of visual stimuli [3]. By comparing experimental conditions in which stimuli are sometimes consciously and sometimes non-consciously perceived, experimenters seek to understand the functional and neural correlates of visual awareness. The answer to the question of the limits of non-conscious processing of information is highly relevant for theories of visual awareness as it spotlights the distinction between conscious and non-conscious processing [4], [5]. At present, however, the role of awareness in the processing of sensory signals is debated. In particular, it remains controversial whether the various basic visual features processed in early vision, such as orientation, color and motion, can be bound into meaningful objects without awareness (for reviews see [6], [7]).

In this study we examine two important aspects of the spatiotemporal binding of distributed features into meaningful objects. First, we investigate the degree to which features belonging to a shared, coherent pattern are spatiotemporally bound together outside of awareness. Indeed, spatiotemporal coherence of features provides one of the most important pieces of evidence that those features belong to the same object [8]. Second, we examine the next step in which coherent ensembles are given meaning based on stored knowledge about the identity of particular patterns. A nice example of this process is the recognition of the specific actions in biological motion patterns made up of point-light displays [9]. Although scrambled versions of these displays may still look somewhat “biological” they no longer map onto a specific recognizable action [10].

To study non-conscious processing of motion stimuli we investigated the conditions that influence the probability that a visual motion stimulus “breaks out” of interocular suppression and into awareness. Typically, the probability of detecting stimulus motion depends on factors such as contrast, speed and duration which constitute the 'physical' energy of motion stimuli [11], [12]. Critically, these aspects of the visual stimulus could, in principle, be processed in individual neurons under binocular suppression, even if the magnitude of responses was reduced [13]. In our experiments we controlled these parameters and manipulated only the coherency of displayed moving dots. Under conditions in which stimuli are visible, increasing coherence should lead to better performance in detection and direction discrimination tasks [14], [15]. However, motion coherence would seem to require spatiotemporal binding across an ensemble of neurons. We employed the breaking continuous flash suppression (b-CFS, [16]) paradigm to assess whether increasing coherence for stimuli that are presented under interocular suppression would improve detection as well.

## Materials and Methods

### Subjects

Eight subjects (six male, two female; age range: 22–33 years) were recruited for Experiment 1, eight subjects (seven male, one female, age range: 24–33) for Experiment 2, and nine subjects (four male, five female; age range: 21–36 years) for Experiment 3. All participants had normal or corrected-to-normal vision. All subjects gave informed written consent according to the guidelines of the University of Trento ethical committee and received course credit for their participation. This study was approved by the ethics committee of the Faculty of Cognitive Sciences of the University of Trento.

### Apparatus

Stimuli were generated using the Matlab Psychtoolbox [17] and displayed on a 21″ Phillips Brilliance 109P4 monitor (1024×768 Pixels; 85 Hz refresh rate; gamma corrected) at a viewing distance of 48 cm. Stimuli were presented against a gray background (CIE coordinates: x = 0.29 y = 0.32 z = 0.39; luminance = 18.2 cd/m^2^). Subjects sat on a height adjustable chair, positioned their head in a chin-rest with a head-bar and viewed stimuli through a mirror stereoscope.

### Stimuli

**Figure 1:**
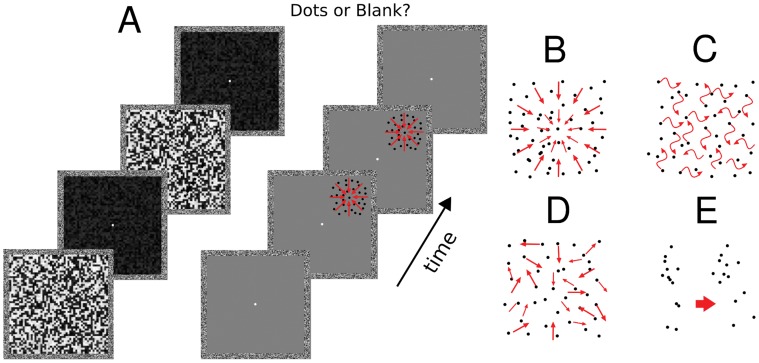
Breaking Continuous Flash Suppression. A- Experimental paradigm: schematic representation of one trial (left panel). Two masks were shown to the left eye of subjects at an alternating rate of 9.45 Hz for 4 s. The pattern of dots was presented to the right eye for variable durations (see methods) and had a random onset time between 0.5 s and 3.5 s after trial start. After 4 s of mask presentation subjects had to indicate with a button press whether they had detected any moving dots or not (2AFC). Four types of motion patterns of dots were used in the experiments (right panel). B- Radial motion, C – Random Walk motion, D- Random trajectories motion, E- Biological motion.

Experiment 1 and experiment 2 comprised three different types of motion stimuli, as described in detail below: radial, random walk or random trajectory. Stimuli were presented inside two square frames measuring 9×9 degrees (one for the left and one for the right eye), with the frame helping to promote stable binocular fusion (Figure 1). On each trial we presented 40 black dots of 3×3 pixels in size, with 13% of Michelson contrast and a speed of 1 deg/sec. Michelson contrast was defined as Lmax – Lmin/Lmax+Lmin, where Lmax and Lmin represent the maximum and minimun luminance. Dots were displayed within an invisible circular aperture of 2.4 degrees of diameter. On each trial the aperture was presented in one of the four quadrants (see Figure 1). The center of each quadrant had an eccentricity of 3 degrees horizontally and vertically from the central fixation dot.

Dots remained in their position for two video frames (∼24 ms), resulting in an effective frame-rate for dot motion of 42.5 Hz. Dots moved for a limited lifetime of ten frames, after which they were reborn in a new random location. On each motion frame 10% of the dots were extinguished and redrawn at a random location inside the aperture. The lifetime of each dot was therefore ∼240 ms (10 motion frames). Dots were redrawn at a random position whenever they would have moved out of the circular aperture or arrived closer than 0.1 degrees from the virtual center of the moving stimuli. Importantly, stimuli in each experimental condition (radial motion, random walk and equal trajectories) shared the same physical parameters. Coordinates on each motion frame were computed on polar coordinates, based on the following set of equations:
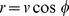


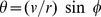
where r is the radial velocity of dots, θ the angular velocity, v is local speed (in degrees over seconds) and Ф defines the type of motion. In the radial condition we used a Ф of 180°, which defines contraction [18], making all dots move towards the center of the aperture. In the random walk condition a random Ф (chosen among a uniform distribution between 0 and 360°) was assigned to each dot on each motion frame. In the case of the random trajectories condition a random Ф (chosen among a uniform distribution between 0 and 360°) was assigned to each dot at the beginning of each trial and remained constant afterwards through all motion frames.

In experiment 3 we presented subjects with point-light displays depicting four different human actions (throwing a ball, punching someone, kicking a ball, kicking someone; for details [10]). For each stimulus, two different viewing angles (lateral to the left/right) were reconstructed. Each action lasted 1.5 s. The noise control stimuli were built by rotating the trajectories of 12 numbers of markers by 90 or 270°. This rotation disrupted local form information while keeping the overall physical stimulation intact: number, contrast and the speed distribution of dots. Under this condition, performance in recognizing actions is at chance [10].

A total of 40 pairs of masks were created for the experiments and one pair was randomly selected for each trial. The masks were 9×9 degrees in size, covering the entire area inside the left eye frame, and consisted of a combination of white, black or gray squares of 0.07 degrees of visual angle (Figure 1). Within each pair of masks, one contained randomly assigned squares in the luminance range of 0–0.3 (where 0 means black pixels; CIE: x = 0.35; y = 0.37; luminance: 0.25 cd/m2). The second mask had exactly the same distribution of squares but 75% of its squares were changed to a luminance range between 0.8–1 (where 1 means white pixels; CIE coordinates: x = 0.28; y = 0.30; luminance: 80 cd/m2). In this way only luminance changes and no first order motion was present from adjacent pixels in the masks.

### Procedures

On each trial the pair of masks was presented to the left eye for 4 s. The masks alternated on the screen every 9 frames (9.45 Hz). Dots were presented to the right eye with a random onset time inside a temporal window between 0.5 s and 3.5 s after trial start (Figure 1). After 4 s subjects had to indicate by button press whether or not they had detected the moving dots (2AFC).

In each experiment we varied the type of motion stimuli that were presented to subjects. In experiment 1 we presented radial motion and random walk motion; in experiment 2 radial motion and random trajectory motion; and in experiment 3 biological motion and biological motion noise control stimuli (see methods). Experiments 1, 2 and 3 presented a total of 428 trials each: 320 trials with dot stimuli and 108 blank trials. An equal number of trials was presented in each of the four quadrants. In each quadrant, there were 40 trials with coherent motion (for experiment 1 and 2: radial motion; for experiment 3: biological motion) and 40 trials with random motion (for experiment 1 random walk motion, for experiment 2 random trajectories motion and for experiment 3 biological motion noise stimuli, see stimuli section). This accounted for 8 trials × 5 durations × 2 motion conditions for each quadrant. The entire session was divided into 4 blocks of 107 trials each, with trials presented in pseudo-random order and with an equal probability of being presented in any of the 4 quadrants. The dots had durations of 100, 200, 400, 800 and 1600 ms for experiments 1 and 2 and 100, 400, 800, 1200 and 1600 ms for experiment 3.

### Statistical Analysis

**Figure 2:**
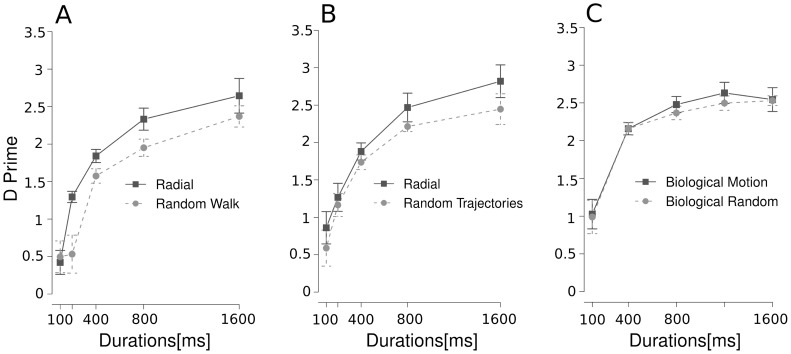
Detection of motion patterns under interocular suppression. For each condition, d' is plotted as a function of dot duration. Patterns of dots moving coherently in radial direction towards the center had a higher detectability than patterns of random walk dots (experiment 1, panel A) or patterns of dots with random trajectories (experiment 2, panel B). A main effect of the type of motion was observed for both conditions. In contrast, meaningful biological motion did not lead to improved detection of suppressed motion over random biological motion (experiment 3, panel C).

To obtain a measurement of the ability of subjects to detect the moving dots we applied signal detection theory [19] and calculated d' values as a dependent variable for each of our experimental conditions (Figure 2). In all experiments stimulus duration and motion coherence were used as factors in repeated-measures and mixed model ANOVAs.

## Results

Dot stimuli with highly coherent radial motion had a higher detection rate than dots with random walk motion (figure 2A, ANOVA, main effect of duration F(4; 28) = 30.83, p<0.001, coherence F(1,7) = 7.23, p<0.05 and an interaction between the factors F(4,28) = 4.27, p<0.01). A similar result was found for random trajectories (figure 2B, main effect for duration F(4; 28) = 19.03, p<0.001 and coherence F(1; 7) = 8.31, p<0.05 but no interaction between the factors F(4; 28) = 0.54, p = 0.70).

To assess whether d' for radial coherent motion differed for experiment 1 and experiment 2 we ran a mixed model ANOVA with stimulus duration as within subject and experiment as between participant variables. This analysis yielded a main effect of stimulus duration F(4,70) = 13.02, p<0.001, but no effect for the factor experiment [F(1,70) <1], nor for an interaction [F(4,70) <1]. To further characterize our results we first computed the mean d' performance on the radial coherent condition for experiments 1 and 2. Second, we tested the difference between the random walk condition in experiment 1 and the equal trajectories condition in experiment 2 against the mean d' values for the two radial conditions. For the random walk condition a mixed model ANOVA with stimulus duration as within subject and coherency as between participant variable revealed a main effect of stimulus duration [F(4,28) = 29.74, p<0.001], and a main effect of coherency [F(1,35) = 14.73, p<0.001], but no interaction [F(1,35) <1]. Similar results were found for the equal trajectories condition: a significant main effect for the factor stimulus duration [F(4,28) = 26.13, p<0.001], and a main effect of coherency [F(1,35) = 4.34, p<0.05], but no interaction was found [F(1,35) <1].

Regarding biological motion stimuli (figure 2C), we did not find a higher detection rate for recognizable biological motion as compared to detection of scrambled motion. Although there was a main effect of duration [F(4; 32) = 26.77, p<0.001], as expected, there was no effect of coherence [F(1; 8) = 0.53, p = 0.48] and no interaction between the factors [F(4; 32) = 0.20, p = 0.93].

Biological motion stimuli and their scrambled controls are characterized by acceleration profiles that are absent in the radial, random walk and random trajectories conditions (where only constant motion is present). However, we could not reject the null hypothesis that the overall mean detection among experiments are equal (between subjects ANOVA, F(2,22) = 0.41, p = 0.66), as would be expected if the acceleration profiles would have played a major role in boosting detection performance. The same held for the analysis on the ceiling performance at 1600 ms duration among experiments (between subjects ANOVA, F(2,22) = 0.71, p = 0.50).

## Discussion

Our main finding is that radial motion coherency facilitates detection of patterns of moving dots presented under interocular suppression. Our results suggest that the visual system is able to extract coherence out of radial non-conscious motion information. Importantly, the use of an unspeeded detection task across the 3 experiments allowed us to avoid any response bias or strategy bias in participants' responses that might be present in b-CFS paradigms that employ reaction times as a dependent measure [16]. D’ is a measure of detection and at the same time an objective measure of conscious access [4], [5]. By definition, visual processes occurring before detection are outside of conscious awareness for the subject [20], [21].

Several studies have shown that motion adaptation can survive interocular suppression and generate motion aftereffects [22]–[24]. Also, the processing of non-conscious features has functional significance as suppressed motion patterns can influence vision during binocular rivalry [3], [25] and perceptual learning [26]. Moreover, neurophysiological studies in monkeys have shown that a proportion of neurons in monkey complex MT/MST fire in response to the physical presentation of motion to the retina, irrespective of the monkey's subjective response [13]. These findings show that the visual system is able to process non-conscious motion information to some extent. However, previous studies have reported that motion processing outside of awareness is confined mainly to simple translational motion which might be encoded in early visual cortex and based on local detectors, rather than requiring the spatiotemporal integration of these signals in other areas such as MT/MST [24], [27]. On the other hand, it is well known from fMRI and psychophysical studies that even though rivalry attenuates visual adaptation to form and motion, the suppressed stimulus is well represented in the dorsal pathways, particularly in hMT+ [28]. Our data is in agreement with this statement.

One way in which CFS may keep a stimulus from breaking through interocular suppression is by reducing its effective strength and thus its exogenous saliency [23]. Many stimulus properties that break through interocular suppression, such as contrast and abrupt onset, would be reduced by repeated spatiotemporal masking, although the stimulus would still be processed by the visual system (leading, for example, to motion aftereffects). Motion coherence provides crucial information about the mechanisms of interocular suppression under CFS by showing that this background processing of visual information not only occurs but that the visual system spatially and temporally integrates visual signals outside of awareness. This would place motion coherence, a relatively “high-level” property requiring integration over time, in the company of other low-level visual features that are processed in the absence of attention or awareness.

In a subsequent experiment we examined whether the meaningfulness of coherent motion patterns would also contribute to the breakthrough from suppression. Previous studies have shown that linear and spiral motion stimuli can be processed in the absence of visual awareness [22]–[24], [27]. To test whether this also applies to biological motion, we presented subjects with stimuli that were perfectly matched in their physical parameters but varied in the property of conveying recognizable biological motion. Our results suggest that adding meaning to biological motion does not help subjects to detect stimuli more often during CFS. On the one hand, this result might seem surprising given the importance for basic survival of a high sensitivity to biological motion. Biological motion has been considered as a bottom-up process by computational models [29] and it has been shown that recognition of biological motion can be rapid [30] and resistant to noise [31]. Most of the motion-selective areas in the cortex are activated by biological motion [32] and some studies have shown specific activation of portions of the STS in the ventral stream [33], [34]. On the other hand, our results suggest that the recognition of meaningful in comparison to scrambled biological motion might depend on additional processes not involved in coherent radial motion processing. Biological motion is elaborated by different areas than radial motion, with a stronger representation along the ventral pathways [33], which presumably makes it more susceptible to suppression during rivalry. Previous studies have suggested the presence of hard-wired dedicated detectors for radial motion [35], while the variety of different types of biological motion may require a more flexible system that maps diverse biological motion patterns onto specific, meaningful actions. It may be the case that the building blocks of biological motion are processed outside of awareness, attracting attention, but that matching the ensemble of biological motion cues to specific actions requires a later step of processing which is mediated by awareness rather than detected automatically.

In order to examine whether our pattern of results are specific to motion processing or, perhaps, reflect a more general principle of visual processing outside of awareness, it is useful to compare our findings with recent studies using faces as stimuli. For face adaptation, shape distortion aftereffects (adaptation to a distorted face biases perception by making the original face to look distorted in a direction opposite to the adapting distortion [36]) can occur under interocular suppression [37], while identity aftereffects appear to be completely abolished under interocular suppression [37]–[39]. Given that shape processing, underlying the distortion aftereffect, involves largely bottom-up features while identity involves assigning identity to the ensemble of features, this nicely parallels the pattern of results found with motion. Overall, motion and face perception show a similar trend in terms of non-conscious processing under inter-ocular suppression, with basic features and some binding of basic features preserved (but reduced in magnitude) but no operations which require assigning meaning (identity) to these bound features.

In summary, our results are in line with the “unconscious binding hypothesis”, which suggests that the visual system “cannot only encode invisible features (orientation, motion direction, etc.) but can also temporally bind distributed invisible features to give rise to cortical representations, though fragile” [6]. The present findings provide further evidence for binding of feature information outside of awareness and extend this to a visual property, motion coherence, which would seem to require coherent and temporally extended neural responses in a fairly high-level visual area. Our findings provide a novel and testable hypothesis about the scope and limits of spatiotemporal binding of distributed features outside of awareness. A deeper understanding of the functional role and the limits of processing non-conscious stimuli would shed light on theories of visual awareness [4], [6] that seek to understand the differences in function and physiology of conscious versus non-conscious processes.
